# Development and clinical utility of a novel diagnostic nystagmus gene panel using targeted next-generation sequencing

**DOI:** 10.1038/ejhg.2017.44

**Published:** 2017-04-05

**Authors:** Mervyn G Thomas, Gail DE Maconachie, Viral Sheth, Rebecca J McLean, Irene Gottlob

**Affiliations:** 1 Ulverscroft Eye Unit, Department of Neuroscience, Psychology and Behaviour, University of Leicester, Leicester, UK

## Abstract

Infantile nystagmus (IN) is a genetically heterogeneous disorder arising from variants of genes expressed within the developing retina and brain. IN presents a diagnostic challenge and patients often undergo numerous investigations. We aimed to develop and assess the utility of a next-generation sequencing (NGS) panel to enhance the diagnosis of IN. We identified 336 genes associated with IN from the literature and OMIM. NimbleGen Human custom array was used to enrich the target genes and sequencing was performed using HiSeq2000. Using reference genome material (NA12878), we show the sensitivity (98.5%) and specificity (99.9%) of the panel. Fifteen patients with familial IN were sequenced using the panel. Two authors were masked to the clinical diagnosis. We identified variants in 12/15 patients in the following genes: *FRMD7* (*n*=3), *CACNA1F* (*n*=2), *TYR* (*n*=5), *CRYBA1* (*n*=1) and *TYRP1* (*n*=1). In 9/12 patients, the clinical diagnosis was consistent with the genetic diagnosis. In 3/12 patients, the results from the genetic diagnoses (*TYR*, *CRYBA1* and *TYRP1* variants) enabled revision of clinical diagnoses. In 3/15 patients, we were unable to determine a genetic diagnosis. In one patient, copy number variation analysis revealed a *FRMD7* deletion. This is the first study establishing the clinical utility of a diagnostic NGS panel for IN. We show that the panel has high sensitivity and specificity. The genetic information from the panel will lead to personalised diagnosis and management of IN and enable accurate genetic counselling. This will allow development of a new clinical care pathway for IN.

## Introduction

Infantile nystagmus (IN) is characterised by the involuntary oscillation of the eyes present at birth or manifesting during infancy. Nystagmus has an estimated prevalence of 2.4 in 1000^1^ and is associated with significant negative social stigma and poor visual function scores.^2, 3^ IN is a genetically heterogeneous disorder that can arise due to variants of genes involved in retinal and brain development. Identifying the underlying cause of IN can be challenging due to the diverse range of genetic disorders associated with it. For example, variants that affect the development of retinal photoreceptors result in achromatopsia, congenital stationary night blindness or retinal dystrophies, all of which are typically associated with IN.^4, 5^ Similarly, variants that cause albinism, Hermansky Pudlak syndrome and aniridia can also present with foveal hypoplasia and IN.^5, 6^ Recently it has been shown that a small proportion of patients with *FRMD7* variants can have foveal hypoplasia and IN.^7^ Similarly, recessive variants of *SLC38A8* are associated with foveal hypoplasia.^8^ Congenital neurological syndromes such as spinocerebellar ataxia, episodic ataxia and structural malformations (eg microcephaly) can also present with IN, however, typically with distinctive features such as cerebellar signs.^9^ Often there is significant overlap in phenotypical characteristics between these disorders. This results in a long differential diagnosis list based on an often challenging clinical examination of a child with nystagmus.

Patients with IN often undergo numerous investigations to identify the underlying cause of the disease. In addition to a paediatric ophthalmological and neurological examination, many patients may be subject to specialised investigations that include electroretinograms (ERGs), visual evoked potentials (VEPs), optical coherence tomography (OCT) and, in some instances, MRI under anaesthesia/sedation for children who cannot cooperate. Previous work has shown that in approximately 40% of cases of IN VEPs were inconclusive in children due to unreliable traces.^10^ Obtaining reliable results from other investigative modalities, such as OCT, are also user-dependent and only available in a few centres for infants and young children. It also requires significant co-operation from the patient, which can be challenging in children.

With the advent of next-generation sequencing (NGS) it is now possible to develop targeted sequencing panels for a specific phenotype. However, to date there have been no studies systematically investigating the use of a targeted NGS panel for IN. Therefore, we aimed to develop a NGS panel to investigate its use in clinical practice, specifically assessing sensitivity, specificity and diagnostic potential of this panel.

## Materials and methods

### Subjects

Fifteen patients with IN were recruited from the University Hospitals of Leicester to take part in this study. Patients were chosen randomly from a database^1^ of 300 familial cases of IN. The inclusion criteria for this study were (a) onset of nystagmus within the first 6 months of life and (b) clinical suspicion of a Mendelian inheritance. Previous work in idiopathic IN (IIN) singletons identified *FRMD7* variants between 0 and 7% of cases, thus suggesting that novel genes or non-genetic factors could be causative in singletons.^11, 12^ Exclusion criteria were acquired nystagmus or evidence of contributory non-genetic aetiological factors for the nystagmus such as retinopathy of prematurity, infections, trauma, drug exposure (eg, opiates, benzodiazepines) *in utero* and fusional maldevelopment nystagmus syndrome. All patients underwent detailed ophthalmic examinations. Eye movement recordings, electrodiagnostic tests and OCT were also obtained where possible. Eye movement recordings were obtained using infrared video pupil tracker (EyeLink II and EyeLink1000, SR Research Ltd, Ontario, Canada). OCTs were obtained using handheld OCT (Bioptigen Envisu system, Durham, NC, USA) and a table-mounted device (SOCT Copernicus HR, OPTOPOL Technology S.A., Zawiercie, Poland). Each clinical diagnosis was based on specialist paediatric ophthalmologist assessment (by IG) and determined prior to sequence analysis. Authors MGT and GDEM performed the sequence analysis and therefore were blinded to the clinical diagnosis and phenotypical characteristics. The clinical characteristics of the patients are shown in Table 1. Written consent was obtained from all patients (or parents in case of children unable to consent). The study adhered to the tenets of the Declaration of Helsinki and was approved by the local ethics committee.

### Nystagmus gene panel development and NGS

Saliva samples were obtained from all patients using the Oragene DNA sample Collection Kit (OG-500, DNA Genotek Inc., Ottawa, Ontario, Canada). DNA was extracted using the Qiagen DNA extraction kits as per the manufacturer's recommendations.

The genomic DNA was randomly fragmented with a base pair peak of 200–250 bp, subsequently adapters were ligated to both ends of the resulting fragments. DNA was amplified by ligation-mediated PCR, purified and hybridised to NimbleGen Human custom array (NimbleGen SeqCap EZ Choice, Roche Nimblegen Inc., Madison, WI, USA) for enrichment. NimbleDesign was used to create the custom probeset design for the nystagmus panel. All known genes associated with IN were included within the panel (*n*=336). The genes were selected based on previous literature, OMIM records (www.omim.org) and RetNet, the Retinal Information Network ^13^ (see Supplementary Table S1). The probes were designed to capture the exons and 40 bases flanking the splice junction. If there were more than one transcript, we chose the longest transcript from the GRCh37/hg19 assembly. We also included known *cis*-regulatory elements associated with nystagmus genes that have previously been described in the literature.^14, 15^ Specific targets included: (1) an ultraconserved *cis*-element located 150 kb downstream from *PAX6*, variants of which has been described in a patient with aniridia and nystagmus (variant: hg19: chr11:g.31685945G>T)^15^ and (2) highly conserved *cis*-elements located up to 11 kb upstream of the *TYR* gene as described by Ray *et al*,^14^ which includes the *TYR* promoter. This region is hypothesised to harbour variants causative of albinism^14^; however, to date no sequencing study has been undertaken to include this region.

Resulting libraries were sequenced on a HiSeq 2000 (Illumina, San Diego, CA, USA) according to the manufacturer's recommendations for paired-end (2 × 100 bp) protocol. The mean coverage depth ranged between 181 × and 301 × (Supplementary Table S2). Percentage of the target sequence covered >15 × was >99% (Supplementary Table S2).

All 15 IN samples and the reference DNA (NA12878—see below) were enriched and sequenced as described above. Allelic variants were reported according to Human Genome Variation Society guidelines.^16^ We identified rare variants by focussing on protein-altering and splice-site changes with an allele frequency of <1% in the 1000 genomes project or in the NHLBI ESP exomes. Variants that were previously established to cause IN were included and classified as pathogenic even if allele frequency was >1%. The allelic variations were also assessed against the sequence data from 70 controls (without nystagmus) and were excluded if present within our control data set. The control cohort consisted of a mixture of ethnicities: European (*n*=52), South Asian (*n*=13), and East Asian (*n*=5).

The variant and phenotype data have been submitted to the Leiden Open Variation Database (http://databases.lovd.nl/shared; individual IDs 00088176–00088188).

### Nystagmus gene panel validation experiment and sequence analysis

The National Institute of Standards and Technology together with the Genome in a Bottle Consortium have developed reference genome material (NA12878 DNA and high-confidence variant calls), which is recommended for assessing variant-call accuracy and understanding biases when developing novel targeted sequencing panels. This will not only consider the efficiency of the capture and sequencing process but also provide a benchmark against which we could assess different alignment and variant-calling algorithms. We obtained the NA12878 DNA (reference DNA) from Coriell Cell repositories. Library construction and sequencing was performed as detailed above.

The read files obtained from sequencing were analysed using the BWA-GATK pipeline. The variants generated were assessed against previously published gold-standard variants for the sample.^17^ This includes a combination of 11 NA12878 whole-human genome data sets and three exome data sets, generated across five sequencing platforms to eliminate bias from any single platform. The high-confidence variant calls are available at: ftp://ftp-trace.ncbi.nlm.nih.gov/giab/ftp/release/NA12878_HG001/.

The reads were aligned using BWA v0.7.5 ^18^ to the reference genome (hg19). Samtools v1.2 ^19^ was used to convert, sort and index the alignment.bam files. Picard v1.93 (http://broadinstitute.github.io/picard/) was used to remove duplicates. GATK v3.4-0 ^20^ was used for local realignment around indels and recalibrate quality scores. Single-nucleotide variants and indels were detected using GATK. Further annotation and filtering was performed using ANNOVAR.^21^ We utilised FishingCNV v2.1 for copy number variation analyses.^22^

We determined the assay sensitivity, specificity and balanced accuracy for the NA12878 sample by computing the true positives (TP), false positives (FP), true negatives (TN) and false negatives (FN). Sensitivity=TP/(TP+FN); specificity=TN/(TN+FP) and balanced accuracy=(sensitivity+specificity)/2.

Sequence analyses were performed on a high-performance computing cluster at the University of Leicester.

## Results

### Diagnostic potential of the nystagmus gene panel

The pedigrees of all the 15 subjects associated with IN are shown in Supplementary Figure S1. The phenotypical characteristics and investigations performed are summarised in Table 1. The significant variants identified are shown in Table 2. Examples of nystagmus waveforms identified on eye movement recordings are shown in Supplementary Figure S2. This shows significant familial variability of nystagmus waveforms.

The masked analyses revealed pathogenic or probable pathogenic variants in 9 out of the 15 patients (Table 2). This was consistent with the clinical diagnosis in six out of the nine patients. A hemizygous missense (c. 796G>C, p.(Ala266Pro)) and frameshift (c.1262delC, p.(Pro421LeufsTer23)) variants of *FRMD7* were identified in NYS-001 and NYS-003, respectively. The clinical features were consistent with a diagnosis of IIN. Both patients had relatively good visual acuity, stereopsis and horizontal nystagmus. In NYS-003 ERGs, VEPs and OCTs were normal. It was not possible to perform further investigations in NYS-001 due to poor cooperation. Hemizygous missense (c.299T>G, p.(Leu100Arg)) and nonsense (c.2905C>T, p.(Arg969Ter)) variants of *CACNA1F* were identified in NYS-002 and NYS-004, respectively; both patients were male subjects with reduced visual acuity and predominantly horizontal nystagmus with a vertical component. ERGs showed the characteristic ‘negative' waveform (a-wave larger than the b-wave) in response to bright flashes after dark adaptation (scotopic conditions), consistent with the clinical diagnosis of congenital stationary night blindness. Significant intrafamilial variability of nystagmus was noted in NYS-002 (Supplementary Figure S2).

**Figure 1:**
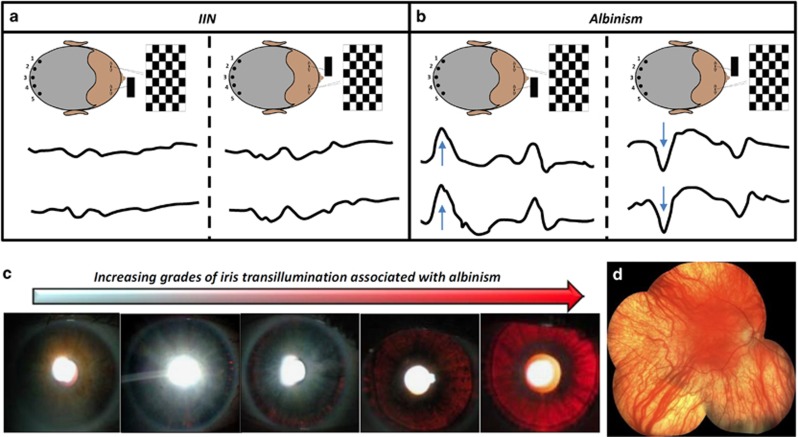
Multichannel VEPs to investigate intracranial visual pathway dysfunction (**a** and **b**). Monocular stimulation is achieved by occluding one eye and presenting a pattern stimulus (checkerboard stimulus). Four lateral electrodes (1 and 2 on left occiput, 4 and 5 on the right occiput) and one midline electrode (electrode 3) are used. Five raw traces corresponding to the polarity of each electrode are obtained during monocular stimulation. In order to improve signal to noise, VEP traces shown are based on the polarity differences between the electrodes placed on the left and right occiput and obtained after averaging. Individuals with a normal visual pathway would not have any significant difference in the polarity between the corresponding lateral electrodes (1 *vs* 5 or 2 *vs* 4). Thus a subtracted waveform (1–5 and 2–4) would show no significant polarity differences as seen in the IIN patient (NYS-007) (**a**). However, in a patient with chiasmal misrouting as seen in albinism (NYS-012) asymmetric responses are seen (blue arrow) (**b**). Different degrees of iris transillumination defects seen with albinism (**c**). Pale fundus associated with albinism (**d**).

**Figure 2:**
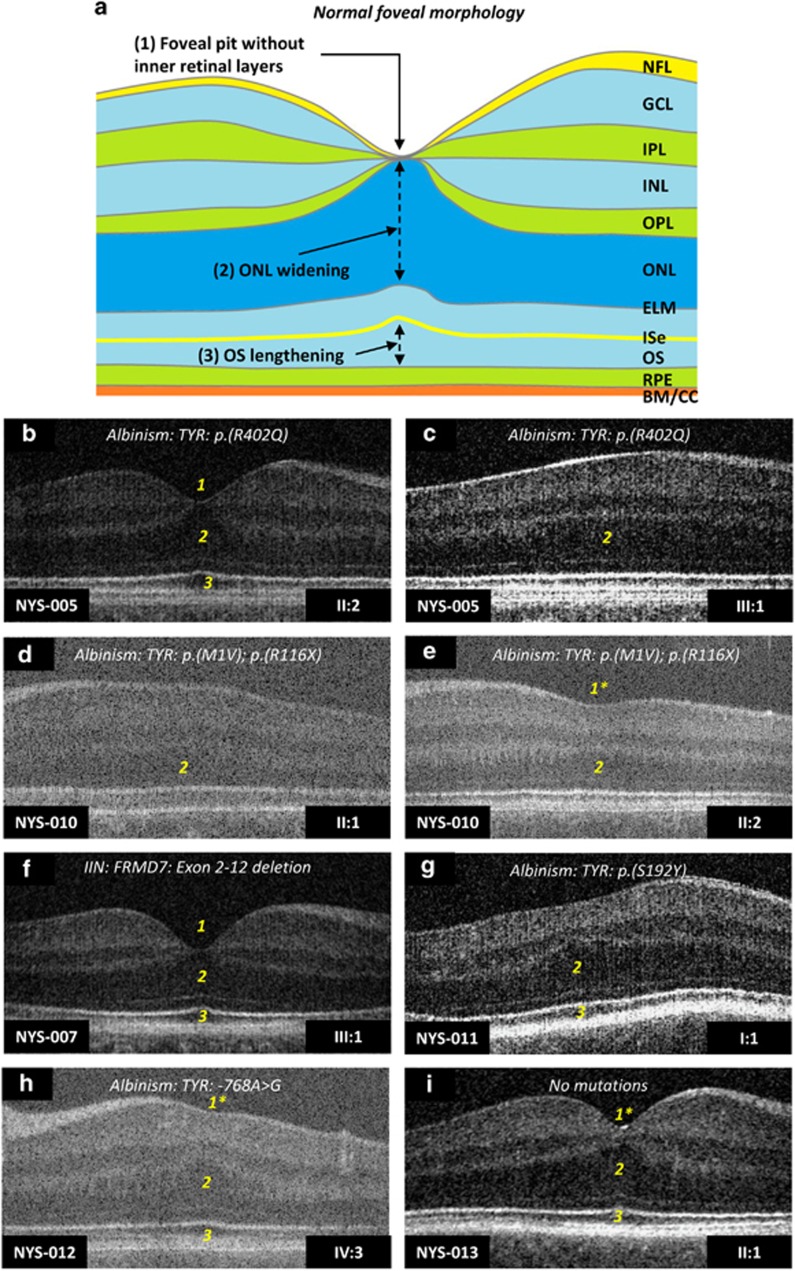
Optical coherence tomograms showing the retinal structure in patients with IN. Figure (**a**) shows an illustration of normal foveal morphology with the features that indicate foveal specialisation which include: (1) deep foveal pit, (2) displacement of inner retinal layers, (3) ONL widening, and (4) OS lengthening. In family NYS-005, the mother (II:2) had a normal foveal structure (**b**) with all four features present (labelled 1–4); however, her son (III:1) did not have a foveal pit and only had ONL widening (**c**). Similarly, in family NYS-010 we observe phenotypical heterogeneity with regards to the foveal structure. Both sisters had compound heterozygous variants of the TYR gene; however, in subject II:1 there was only ONL widening, whereas in subject II:2 there was both ONL widening and a rudimentary foveal pit (denoted by 1*). In family NYS-007, OCT from subject III:1 with FRMD7 deletions has normal foveal morphology. Subject I:1 in family NYS-011 with albinism has no foveal pit but has ONL widening and OS lengthening. In family NYS-012 with albinism, we observe foveal hypoplasia with a rudimentary foveal pit (1*) in subject IV:3, as well as ONL widening and OS lengthening. In family NYS-013, we were unable to identify any variants that affect function within the known nystagmus genes; however, subject II:1 was noted to have subtle foveal hypoplasia with a shallow foveal pit (1*) and persistence of inner retinal layers posterior to the fovea. The numbers (1–4) within the tomograms represents the presence of the foveal specialisation, with 1* representing a shallow pit in foveal hypoplasia. Abbreviations: NFL, nerve fibre layer; GCL, ganglion cell layer; IPL, inner plexiform layer; INL, inner nuclear layer; OPL, outer plexiform layer; ONL, outer nuclear layer; ELM, external limiting membrane; ISe, inner segment ellipsoid; OS, outer segment; RPE, retinal pigment epithelium; BM/CC, Bruchs membrane/choriocapillaries.

Compound heterozygous variants of *TYR* were identified in NYS-010 (c.1A>G, p.(Met1Val) and c.346C>T, p.(Arg116Ter)) and NYS-015 (c.823G>T, p.(Val275Phe) and c.1205G>A, p.(Arg402Gln)). Both patients had significantly reduced visual acuity, no detectable stereopsis, hypopigmentation of the skin, trans-illumination defects of the iris, horizontal nystagmus and foveal hypoplasia. Multichannel VEPs showed asymmetry of hemispheric responses on monocular stimulation indicating chiasmal misrouting (Figure 1). The phenotypical features were consistent with a clinical diagnosis of albinism. The location of the amino-acid changes in relation to the protein are shown in Supplementary Figure S3. Foveal hypoplasia identified using OCT is shown in Figure 2. Significant intrafamilial variability in foveal morphology was noted in family NYS-011.

**Figure 3:**
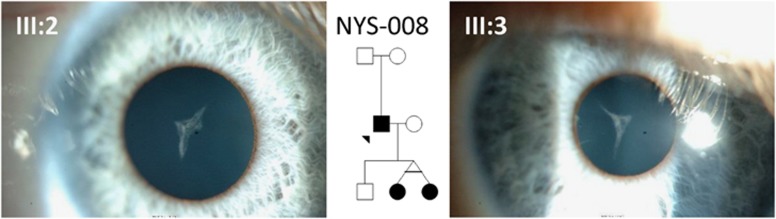
Congenital cataracts associated with CRYBA1 variant in NYS-008. The two images from subjects III:2 and III:3 show (monozygotic twins) sutural cataracts, seen as the opacification of the Y-suture of the foetal nucleus of the lens. Subject II:1 was pseudophakic.

In three out of the nine patients, the genetic diagnosis allowed us to resolve the clinical diagnosis. The clinical diagnosis in NYS-005 was unclear as the phenotype was very mild with good visual acuity, end point nystagmus, no iris trans-illumination defects, normal electrodiagnostic studies and normal retinal morphology on OCT. However, there was a family history of albinism and therefore the working clinical diagnoses included ocular albinism or a carrier for albinism. The eye movement recordings showed variable nystagmus in the family with subjects III:1 and III:2 having pseudopendular nystagmus with foveating saccades while subject II:2 did not have any nystagmus on central fixation but had square wave jerks (Supplementary Figure S2). We identified a homozygous missense variant (c.1205G>A, p.(Arg402Gln)) in *TYR*. In NYS-008, a possible *PAX6*-related phenotype was considered as the clinical diagnosis based on the history of congenital cataracts, ptosis, trans-illumination defects of the iris, reduced visual acuity and nystagmus. Sutural cataracts seen as the opacification of the Y-suture of the fetal nucleus were identified in both of his daughters (Figure 3). Interestingly, two variants were identified: the first was a novel heterozygous variant in *CRYBA1* (c.594G>A, p.(Trp198Ter)), and the second was a novel heterozygous variant in *COL11A1* (c. 2956C>A, p.(Pro986Thr). There was no myopia or history of retinal detachment, suggesting the variant in *CRYBA1* was causative of the phenotype. In NYS-009, the clinical diagnosis of IIN was established based on normal iris, electrodiagnostic tests and OCTs but visual acuity was lower than expected. However, sequence analysis revealed a homozygous missense (c.1579G>C, p.(Glu527Gln)) variant in *TYRP1* consistent with albinism.

Initially, in 6 out of the 15 patients, we did not identify the causative variant based on the masked analysis. However, unmasked to the clinical information we re-analysed the sequence data. In NYS-007, the phenotype was consistent with IIN with an X-linked pedigree. The copy number variation analysis performed using FishingCNV revealed a heterozygous deletion of the *FRMD7* gene. Multiplex Ligation-dependent Probe Amplification (MLPA) analysis showed a large deletion of *FRMD7* exons 2–12. In NYS-011, a previously filtered variant (c.575C>A, p.(Ser192Tyr)) was identified in *TYR.* The variant was filtered out owing to the preset threshold of allele frequency <1%. The exact pathogenicity of the variant is yet to be determined, but there is some evidence that there is reduced enzymatic activity of tyrosine hydroxylase and DOPA oxidase when the Tyr192 variant allele was compared with the Ser192 wild-type allele.^23^ In NYS-012, we identified a homozygous genomic variant (hg19 chr11:g. 88910354A>G) upstream from the transcription start site for the *TYR* gene (c.-768A>G; Supplementary Figure S3). This was a rare variant that has previously not been reported in the literature or in control databases (1000 genomes project, NHLBI ESP exomes or Exomes Aggregation Consortium (ExAC)). We suspect this is likely to affect function as it is located within a GA complex repeat region hypothesised to regulate the transcriptional efficiency of TYR.^14^ In the remaining 3 out of 15 patients (NYS-006, NYS-013 and NYS-014), we were not able to identify a variant responsible for the phenotype.

### Nystagmus gene panel validation experiment

The variants identified following sequencing the NA12878 sample were compared against the previously published gold-standard variants for the sample to determine sensitivity and specificity. We identified a total of 1362 TP. This represents the called variants with a corresponding position in the reference variant file (VCF). A total of 511 FP were identified that represents the called variants without a corresponding position in the reference VCF. A total of 2 241 377 TN were identified. This represents the called reference bases without a corresponding position in the reference VCF. A total of 20 FN were identified. This represents the called reference bases with a corresponding position in the reference VCF. The validation experiment shows a sensitivity of 98.5% and a specificity of 99.9%. The balanced accuracy from this study was 99.2%.

## Discussion

We describe, for the first time, the clinical utility of a NGS panel for childhood nystagmus. This was achieved by designing a customised target enrichment kit for all known genes associated with IN. We analysed 15 patients with IN to assess the ability of this testing strategy in precisely determining the genetic cause of this disorder. We also tested one control subject (NA12878) to determine the assay sensitivity and specificity. During the masked analysis, we had a significant detection rate (9 out of the 15 patients) of variants definitely or probably responsible for the phenotype. The detection rate improved further (12 out of the 15 patients) with the clinical information and a targeted re-analysis of relevant genes. This highlights that even with masked analysis we can identify the genetic cause of most forms of IN. Among the 12 patients, we report two *TYR* variants (c.-768A>G and c.575C>A) that requires further study. In the validation experiment, we show high levels of assay sensitivity (98.5%), specificity (99.9%) and balanced accuracy (99.2%).

**Figure 4:**
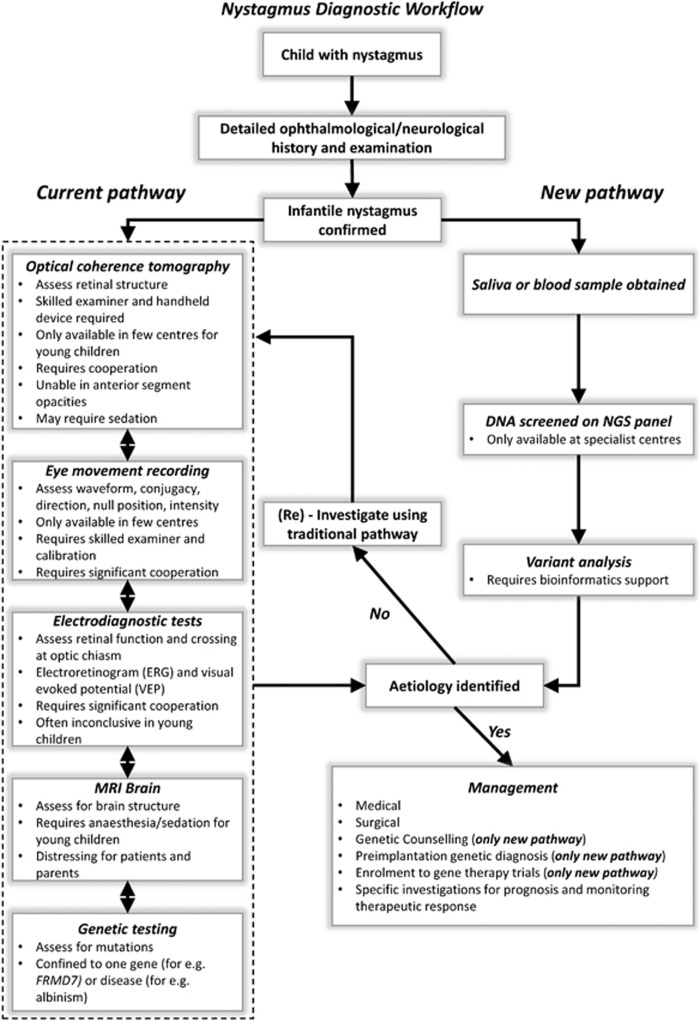
Diagnostic workflow for patients with nystagmus. In the current pathway, patients would undergo a number of different investigations prior to being able to identify the underlying aetiology. The order of performing investigations vary between centres, depends on the clinical context, patient cooperation and availability. Some of the limitations associated with each investigative modality is shown in the box. Not all patients would undergo MRI scans; however, it is one of the investigations of choice for specific forms of nystagmus (for eg, vertical nystagmus) or the presence of additional neurological features such as ataxia were noted. The new pathway that involves using NGS as a frontline diagnostic tool would help establish a genetic diagnosis and thus guide further investigations and targeted treatment.

Though cost and access to screening is frequently an issue, NGS has replaced traditional Sanger sequencing in many clinical settings as the frontline diagnostic tool even prior to performing investigations.^24^ This allows for prompt and accurate diagnosis followed by relevant surgical or medical management. For genetically heterogeneous disorders, such as IN, the use of NGS is more cost effective than Sanger sequencing. It also has the potential to obviate the use of other investigations such as electrodiagnostic testing and neuroimaging, therefore reaching an accurate diagnosis coupled with appropriate genetic information relevant not only to the proband but also to other family members. In children with poor cooperation, electrodiagnostic testing can be unreliable and difficult to perform.^10^ Some investigations are also only available in specialist centres. The results from our blinded analyses from this study indicates that NGS has a role as a frontline diagnostic tool and therefore transforming the diagnostic pathway for patients with IN (Figure 4). It is important to emphasise that NGS is not a replacement for good clinical work up. Therefore, certain investigations would still be relevant based on the clinical context. For example, OCT in foveal hypoplasia also helps provide visual prognostic information based on the grade of foveal hypoplasia.^6^ Similarly, serial eye movement recordings might be necessary to provide an objective measurement of therapeutic response to medical or surgical intervention.

One of the drawbacks of NGS analysis is difficulty detecting copy number variations, yet we demonstrate in this study that we could detect a large *FRMD7* deletion in a family with X-linked IIN where the initial analysis failed to identify this heterozygous deletion. During the masked analysis, the authors did not perform a CNV analysis; however, based on the strong clinical suspicion of X-linked IIN the authors performed a CNV analysis on the NGS data using FishingCNV v2.1,^22^ which revealed a large *FRMD7* deletion. This was confirmed using MLPA analysis. No further CNVs were identified in the other families.

In three patients (NYS-005, NYS-008 and NYS-009), the genetic diagnosis helped resolve the clinical diagnosis. In patient NYS-005, we identified a variant (rs1126809) in *TYR* resulting in the amino-acid substitution p.(Arg402Gln). There has been some debate regarding the pathogenicity of this variant. Oetting *et al*^25^ reported that the R402Q variant is not associated with autosomal-recessive ocular albinism but postulated that a causative variant might be in genetic disequilibrium with the p.(Arg402Gln) variant. However, Chiang *et al*^26^ showed that this allele is strongly associated with albinism patients who have only 1 variant in *TYR*. Berson *et al*^27^ showed that the p.(Arg402Gln) variant resulted in a defect in protein folding, which prevents exit from the endoplasmic reticulum thus resulting in an abnormal phenotype. In patient NYS-009, with clinical diagnosis of IIN but with genetic diagnosis of *TYRP1*, interestingly we noted both horizontal and vertical nystagmus. Previously, a small vertical component to the nystagmus has been described with both *FRMD7* variants and albinism.^28^ However, in this patient the amplitude of the vertical and horizontal components was similar that was atypical.

Patient NYS-008 was originally thought to have a *PAX6*-related phenotype; however, we identified two novel variants in *CRYBA1* and *COL11A1* resulting in the amino-acid substitution p.(Trp198Ter)and p.(Pro986Thr), respectively. Both variants segregated with the phenotype. This highlights the complexity in formulating a diagnosis in patients with IN. Although the patient had congenital cataract, he also had additional features that included ptosis, trans-illumination defects of the iris, reduced visual acuity and nystagmus, therefore a diagnosis of *PAX6*-related phenotype was considered. *COL11A1* variants have been described in association with Stickler/Marshall syndrome.^29, 30^ The patient did not have the classic features of Stickler/Marshall syndrome such as high myopia, retinal detachment and mid facial hypoplasia. Previously, the amino-acid substitution p.(Gly988Val) in *COL11A1* has been described^29, 30^; this is very close to the variant p.(Pro986Thr) that we have reported. Interestingly, the phenotype with the G988V variant was also milder, not associated with retinal detachments; however, cataract and high myopia were present. Nystagmus has previously been described to be associated with *COL11A1* variant.^31^ However, as there was a truncating variant of *CRYBA1* we suspect this is most likely the cause of the phenotype. Previously, nystagmus has been described to be associated with congenital cataracts due to *CRYBA1* variants.^32^ However, the additional phenotypical features such as ptosis and iris transillumination defects have not been documented.

To date, there have been no comparisons between diagnostic ability of exome sequencing and targeted NGS panels in nystagmus. The main advantages of the targeted NGS panel over exome sequencing are: (1) cheaper cost and (2) ability to sequence genomic regions not included or poorly covered within the exome target regions. One of the unique features of the nystagmus NGS panel is that it not only captures the coding sequence along with 40 bases flanking the splice junction but also *cis*-regulatory elements associated with nystagmus genes and deep intronic regions that have previously been described in the literature.^7, 14, 15^ These are genomic regions that could potentially be missed during whole-exome sequencing. For example, previously we have identified deep intronic variants in the *FRMD7* gene (c.285-118C>T) predicted to activate a cryptic splice donor^7^; using an exome sequencing approach, we would have missed these variants. However, in the panel described here we have included these additional sites associated with IN. Similarly, we included regulatory sites upstream from the *TYR* gene. This has allowed us to identify a novel variant upstream from the transcription start site for *TYR* within the GA complex repeat region hypothesised to regulate transcriptional efficiency.^14^ The importance of clinical context was highlighted in subject NYS-011; in our masked analysis, we did not identify any variants likely to affect function. However, her phenotype consisted of (1) asymmetric VEPs suggestive of abnormal decussation of axons at the optic chiasm and (2) trans-illumination defects of the iris, both features of albinism. We, therefore, reviewed all variants within the albinism-related genes and genes associated with optic nerve misrouting.^8^ We identified a previously filtered homozygous *TYR* variant resulting in the amino-acid substitution p.(Ser192Tyr). The variant was filtered out owing to our preset threshold of filtering variants with a minor allele frequency >1%. Previous work has suggested that this variant is associated with variation in skin pigmentation^33^; however, the effects of a homozygous variant is unclear. The Tyr192 variant allele was associated with reduced enzymatic activity of tyrosine hydroxylase and DOPA oxidase when compared with the Ser192 wild-type allele.^23^ Further functional work is necessary to understand the exact effects of both these variants on transcription and translation of *TYR*.

In three patients (NYS-006, NYS-013, NYS-014), we did not identify any significant variants within all known nystagmus-related genes. This is unlikely to be due to insufficient depth of coverage as all three samples had an average coverage depth >180 ×. It is possible that these families harbour variants within novel genes associated with nystagmus or within other *cis*-regulatory elements that are yet to be identified. Therefore, in these families, it would be appropriate to perform whole-genome sequencing to identify the genetic basis of their nystagmus. This study has also highlighted several atypical features that can be encountered such as vertical nystagmus (NYS-009) and a normal retinal structure on OCT (NYS-011) associated with albinism. Hence, the phenotypic spectrum associated with these disorders is larger than we had previously thought, and as NGS testing becomes more accessible, we will be able to identify more genotype–phenotype correlations.

In conclusion, this study shows that NGS has an important role in early screening and diagnosis of patients with IN. It has the potential to complement other investigations currently used for diagnostic purposes and reveal atypical phenotypes associated with certain genes. We demonstrate that the genetic diagnosis was used to revise the clinical diagnosis. Thus it will have a role in the development of an individualised care plan and identifying the genetic basis of phenotypes with atypical features. Moreover, the genetic information enables the accurate genetic counselling and further family planning. With the advent of personalised genomic medicine, the use of the nystagmus NGS panel is a cost-effective frontline diagnostic tool.

## Figures and Tables

**Table 1:** Clinical characteristics of patients with nystagmus

*ID*	*Clinical diagnosis*	*Age*	*BCVA*	*Refraction*		*Colour vision*	*Strabismus*	*Stereopsis*	*AHP*	*Nystagmus characteristics*	*TID*	*Fundus*	*ERG*	*VEP*	*OCT*	*Additional phenotypical characteristics*
				*RE*	*LE*											
NYS-001	IIN	5	0.20	0	0	N	N	85"	Nil	HJ	Nil	N	NP	NP	NP	Nil
NYS-002	CSNB	27	0.48	0	0	N	N	300''	Present	Rotary	Nil	N	Negative waveform (scotopic)	N	N	Nil
NYS-003	IIN	58	0.18	−0.75	0.125	N	N	150''	Present	HJ with PAN	Nil	N	N	N	N	Nil
NYS-004	CSNB	46	0.30	−2.25	−2.25	N	N	85''	Present	HVJ	Nil	N	Negative waveform (scotopic)	N	N	Nil
NYS-005	Ocular albinism/carrier	49	0.00	0	0	N	N	85"	Nil	End point	Nil	N	N	N	N	Family history of albinism
NYS-006	*PAX6*-related phenotype	8	0.25	0	0	NP	ET	600''	Nil	VJ	Nil	N	NP	NP	N	Nil
NYS-007	IIN	30	0.18	−5.00	−4.25	N	N	85''	Present	HJ	Nil	N	N	N	N	Nil
NYS-008	*PAX6*-related phenotype	47	0.48	0	0	N	Hypertropia	Nil	Present	HVJ	Present	N	N	N	N	Congenital cataracts and ptosis
NYS-009	IIN	5	0.775	0	0	N	ET	Nil	Present	HVJ	Nil	N	N	N	N	Nil
NYS-010	Albinism	4	0.70	0.50	0.50	NP	XT	Nil	Present	HJ with PAN	Present	FH	N	Asymmetry	FH	Pale skin and photophobia
NYS-011	Albinism	5	0.325	−3.25	−2.75	N	N	85''	Present	HJ	Present	N	N	Asymmetry	N	Nil
NYS-012	Albinism	18	0.48	6.75	6.625	N	XT	Nil	Present	HJ	Present	N	N	Asymmetry	FH	Nil
NYS-013	*PAX6*-related phenotype	34	0.18	0	0	N	ET	Nil	Present	HJ	Present	FH	N	Inconclusive	FH	Congenital cataracts and eccentric pupil
NYS-014	IIN	1	0.64	3.25	3.25	NP	N	Fusion	Nil	HJ	Nil	N	NP	NP	N	Nil
NYS-015	Albinism	29	0.48	−2.25	−1.75	N	ET	Nil	Present	HJ	Nil	N	N	Asymmetry	FH	Photophobia

Abbreviations: A, abnormal; AHP, anomalous head posture; BCVA, best corrected visual acuity (both eyes); CSNB, congenital stationary night blindness; ERG, electroretinogram; ET, esotropia; FH, fundus hypopigmentation (fundus column); FH, foveal hypoplasia (OCT column); HJ, horizontal jerk; HVJ, horizontal and vertical jerk; IIN, idiopathic infantile nystagmus; LE, left eye; N, normal; NP, not performed due to poor cooperation; OCT, optical coherence tomography; PAN, periodic alternating nystagmus; RE, right eye; TID, trans-illumination defects of iris; VEP, visual evoked potentials; VJ, vertical jerk; XT, exotropia.

Units: Age – years; Visual acuity – LogMAR; refraction – spherical equivalent.

**Table 2:** Variants identified in families with nystagmus

*ID*	*Clinical diagnosis*	*Masked genetic diagnosis*	*Unmasked genetic diagnosis*	*Mutation*	*Mutation type*	*In silico prediction (MutationTaster)*	*In silico prediction (Polyphen2)*	*In silico prediction (CADD score)*	*Exon*	*Accession ID for transcript*	*Accession ID for gene*	*Zygosity*
NYS-001	IIN	*FRMD7*	*FRMD7*	c.796G>C:p.(Ala266Pro)	Missense	Disease causing	Damaging	25.4	Exon 9	NM_194277.2	NG_012347.1	Hemizygous
NYS-002	CSNB	*CACNA1F*	*CACNA1F*	c.299T>G: p.(Leu100Arg)	Missense	Disease causing	Damaging	26	Exon 3	NM_005183.2	NG_009095.2	Hemizygous
NYS-003	IIN	*FRMD7*	*FRMD7*	c.1262delC: p.(Pro421LeufsTer23)	Deletion	NA	NA	NA	Exon 12	NM_194277.2	NG_012347.1	Hemizygous
NYS-004	CSNB	*CACNA1F*	*CACNA1F*	c.2905C>T: p.(Arg969Ter)	Nonsense	NA	NA	NA	Exon 24	NM_005183.2	NG_009095.2	Hemizygous
NYS-005	Ocular Albinism	*TYR*	*TYR*	c.1205G>A: p.(Arg402Gln)	Missense	Polymorphism	Damaging	34	Exon 4	NM_000372.4	NG_008748.1	Homozygous
NYS-006	*PAX6*-related phenotype	Unknown	Unknown	None	NA	NA	NA	NA	NA	NA	NA	NA
NYS-007	IIN	Unknown	*FRMD7*	c.(235+1_236-1)_(*3202_?)del	Deletion	NA	NA	NA	Exons 2–12	NM_194277.2	NG_012347.1	Heterozygous
NYS-008	*PAX6*-related phenotype	*CRYBA1*	*CRYBA1*	c.594G>A:p.(Trp198Ter)	Nonsense	NA	NA	NA	Exon 6	NM_005208.4	NG_008037.1	Heterozygous
NYS-009	IIN	*TYRP1*	*TYRP1*	c.1579G>C:p.(Glu527Gln)	Missense	Disease causing	Benign	8.7	Exon 8	NM_000550.2	NG_011705.1	Homozygous
NYS-010	Albinism	*TYR*	*TYR*	c.1A>G:p.(Met1Val) and c.346C>T:p.(Arg116Ter)	Missense and nonsense	Disease causing	Possibly damaging	23.1	Exon 1	NM_000372.4	NG_008748.1	Compound heterozygous
NYS-011	Albinism	Unknown	Possible *TYR*	c.575C>A:p.(Ser192Tyr)^a^	Missense	Polymorphism	Damaging	25	Exon 1	NM_000372.4	NG_008748.1	Homozygous
NYS-012	Albinism	Unknown	*TYR*	c.-768A>G	Regulatory	NA	NA	NA	Upstream	NM_000372.4	NG_008748.1	Homozygous
NYS-013	*PAX6*-related phenotype	Unknown	Unknown	None	NA	NA	NA	NA	NA	NA	NA	NA
NYS-014	IIN	Unknown	Unknown	None	NA	NA	NA	NA	NA	NA	NA	NA
NYS-015	Albinism	*TYR*	*TYR*	c.823G>T:p.(Val275Phe) and c.1205G>A:p.(Arg402Gln)	Missense	Disease causing; polymorphism	Possibly damaging; damaging	22.6; 34	Exons 2 and 4	NM_000372.4	NG_008748.1	Compound heterozygous

Abbreviation: NA, not applicable. Variant segregation was not assessed in NYS-007 owing to unavailability of other family members.

a Pathogenicity unclear.
