# Supplementary material for: Development and clinical utility of a novel diagnostic nystagmus gene panel using targeted next-generation sequencing
Source: Eur J Hum Genet. 2017 Apr 5;25(6):725–34. doi: 10.1038/ejhg.2017.44 (PMC5477371; doi:10.1038/ejhg.2017.44)
Supplement: Supplementary Table S2 [file ejhg201744x1.docx]

**Supplementary table 2:** Mapping and coverage statistics from targeted next generation sequencing
