# Supplementary material for: Development and clinical utility of a novel diagnostic nystagmus gene panel using targeted next-generation sequencing
Source: Eur J Hum Genet. 2017 Apr 5;25(6):725–34. doi: 10.1038/ejhg.2017.44 (PMC5477371; doi:10.1038/ejhg.2017.44)
Supplement: Supplementary Table S1 [file ejhg201744x2.pdf]

ID	*Gene name*
1	ABCA4
2	ABHD12
3	ACO2
4	ADAM9
5	ADAMTS10
6	ADAMTSL4
7	AFG3L2
8	AGK
9	AGPS
10	AHI1
11	AIMP1
12	AIPL1
13	AKR1E2
14	ALDH18A1
15	ALMS1
16	ANO10
17	AP3B1
18	ARL2BP
19	ARL6
20	ATCAY
21	ATOH7
22	ATP1A2
23	ATP2B3
24	ATXN1
25	ATXN10
26	ATXN3
27	ATXN7
28	B3GLCT
29	BBIP1
30	BBS1
31	BBS10
32	BBS12
33	BBS2
34	BBS4
35	BBS5
36	BBS7
37	BBS9
38	BCOR
39	BEST1
40	BFSP1
41	BFSP2
42	BLOC1S3
43	BLOC1S6
44	C10orf11
45	C2orf71
46	C8orf37

47	CA4
48	CABP4
49	CACNA1A
50	CACNA1F
51	CACNA2D4
52	CACNB4
53	CAPN15
54	CBS
55	CC2D2A
56	CCDC28B
57	CDH23
58	CDH3
59	CDHR1
60	CEP164
61	CEP290
62	CERKL
63	CHM
64	CHMP4B
65	CIB2
66	CLRN1
67	CNGA1
68	CNGA3
69	CNGB1
70	CNGB3
71	CNNM4
72	COL11A1
73	COL18A1
74	COL2A1
75	COL4A1
76	COL9A1
77	CRB1
78	CRX
79	CRYAA
80	CRYAB
81	CRYBA1
82	CRYBA4
83	CRYBB1
84	CRYBB2
85	CRYBB3
86	CRYGC
87	CRYGD
88	CRYGS
89	CYP27A1
90	CYP4V2
91	CYP51A1
92	DHCR7
93	DHDDS

94	DTNBP1
95	EEF2
96	ELOVL4
97	ELP4
98	EPHA2
99	ERCC2
100	ERCC3
101	ERCC6
102	ERCC8
103	EXOSC3
104	EYA1
105	EYS
106	FAM126A
107	FAM161A
108	FBN1
109	FGF14
110	FKRP
111	FKTN
112	FLVCR1
113	FOXC1
114	FOXD3
115	FOXE3
116	FRMD7
117	FSCN2
118	FTL
119	FYCO1
120	FZD4
121	GALK1
122	GALT
123	GCNT2
124	GDF3
125	GDF6
126	GJA1
127	GJA3
128	GJA8
129	GJC2
130	GNAT1
131	GNAT2
132	GNPAT
133	GPR143
134	GPR179
135	GPR98
136	GRM1
137	GRM6
138	GUCA1A
139	GUCA1B
140	GUCY2D

141	HARS
142	HMX1
143	HPS1
144	HPS3
145	HPS4
146	HPS5
147	HPS6
148	HSD17B4
149	HSF4
150	IDH3B
151	IMPDH1
152	IMPG2
153	INPP5E
154	IQCB1
155	ITM2B
156	ITPR1
157	JAM3
158	KCNA1
159	KCNC3
160	KCND3
161	KCNJ13
162	KCNV2
163	KIF11
164	KLHL7
165	LARGE
166	LCA5
167	LMX1B
168	LRAT
169	LRIT3
170	LRP5
171	LTBP2
172	LYST
173	LZTFL1
174	MAF
175	MAK
176	MAN2B1
177	MCPH1
178	MERTK
179	MFRP
180	MFSD6L
181	MIP
182	MIR184
183	MITF
184	MKKS
185	MKS1
186	MTPAP
187	MYH9

188	MYO5A
189	MYO7A
190	NDP
191	NECTIN3
192	NEK2
193	NF2
194	NHS
195	NMNAT1
196	NPHP1
197	NPHP4
198	NR2E3
199	NRL
200	NYX
201	OCA2
202	OCRL
203	OFD1
204	OPA1
205	OPA3
206	OPN1LW
207	OPN1MW
208	OTX2
209	PAX2
210	PAX6
211	PCDH15
212	PDE6A
213	PDE6B
214	PDE6C
215	PDE6G
216	PDE6H
217	PDZD7
218	PEX1
219	PEX10
220	PEX11B
221	PEX12
222	PEX13
223	PEX14
224	PEX16
225	PEX19
226	PEX2
227	PEX26
228	PEX3
229	PEX5L
230	PEX6
231	PEX7
232	PGK1
233	PHYH
234	PITPNM3

235	PITX2
236	PITX3
237	PLP1
238	POMT1
239	POMT2
240	PRCD
241	PRKCG
242	PROM1
243	PRPF3
244	PRPF31
245	PRPF6
246	PRPF8
247	PRPH2
248	PXDN
249	RAB18
250	RAB27A
251	RAB28
252	RAB3GAP1
253	RAB3GAP2
254	RAX2
255	RBP3
256	RBP4
257	RD3
258	RDH12
259	RECQL4
260	RGR
261	RHO
262	RIMS1
263	RLBP1
264	RNLS
265	ROBO3
266	ROM1
267	RP1
268	RP1L1
269	RP2
270	RP9
271	RPE65
272	RPGR
273	RPGRIP1
274	RPGRIP1L
275	RS1
276	SAG
277	SC5D
278	SDCCAG8
279	SEC23A
280	SEMA4A
281	SETX

282	SHH
283	SIL1
284	SIX5
285	SIX6
286	SLC16A12
287	SLC16A2
288	SLC1A3
289	SLC24A1
290	SLC24A5
291	SLC2A1
292	SLC33A1
293	SLC38A8
294	SLC45A2
295	SLC52A2
296	SNRNP200
297	SOX10
298	SOX2
299	SPATA7
300	SPG7
301	SPTBN2
302	SRD5A3
303	SREBF2
304	SYT14
305	TBP
306	TDRD7
307	TFAP2A
308	TMEM114
309	TMEM126A
310	TMEM216
311	TMEM237
312	TMEM67
313	TMEM70
314	TOPORS
315	TRIM32
316	TRPM1
317	TTBK2
318	TTC8
319	TUBGCP6
320	TULP1
321	TYR
322	TYRP1
323	UCHL1
324	USH1C
325	USH1G
326	USH2A
327	VCAN
328	VIM

329	VLDLR
330	VSX2
331	WDPCP
332	WDR19
333	WFS1
334	WRN
335	ZNF423
336	ZNF513
