# Supplementary figures and images for: Development and clinical utility of a novel diagnostic nystagmus gene panel using targeted next-generation sequencing
Source: Eur J Hum Genet. 2017 Apr 5;25(6):725–34. doi: 10.1038/ejhg.2017.44 (PMC5477371; doi:10.1038/ejhg.2017.44)

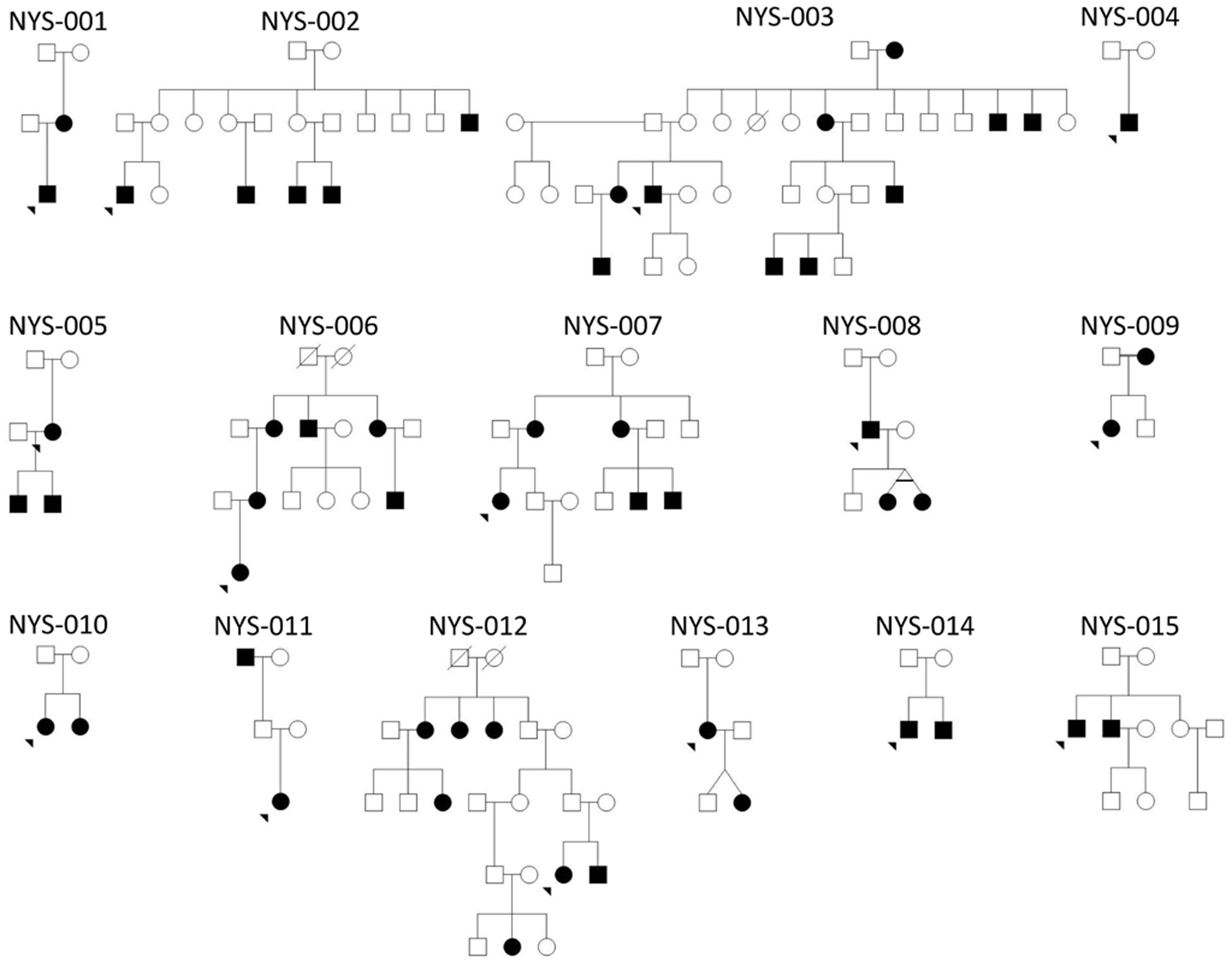

Supplement: Supplementary Figure S1 [file ejhg201744x3.tif]

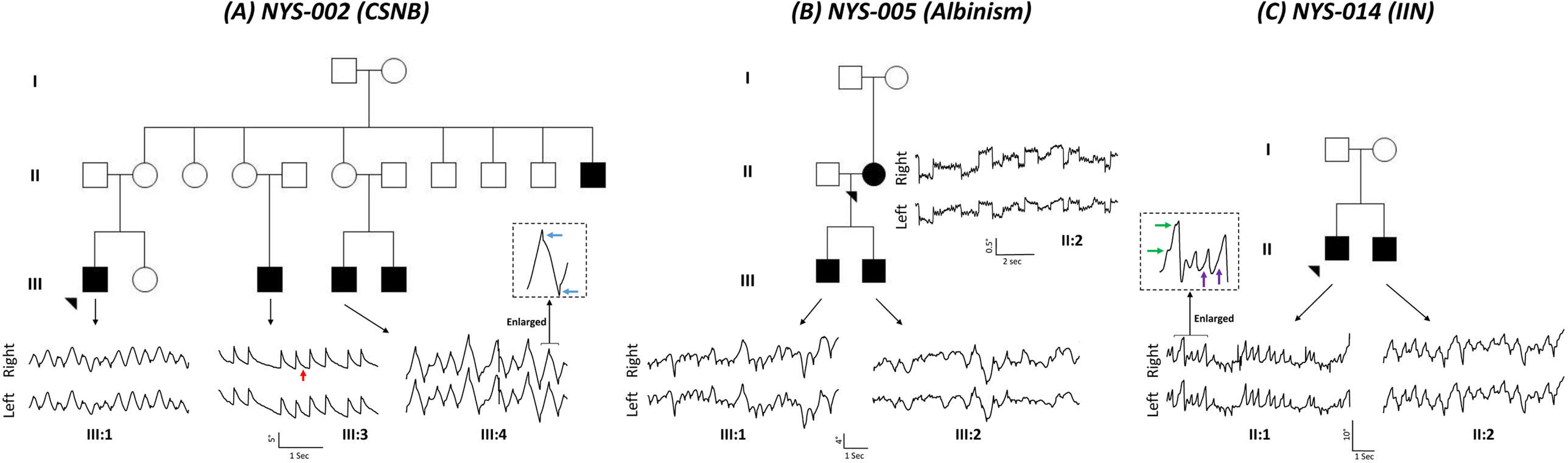

Supplement: Supplementary Figure S2 [file ejhg201744x4.tif]

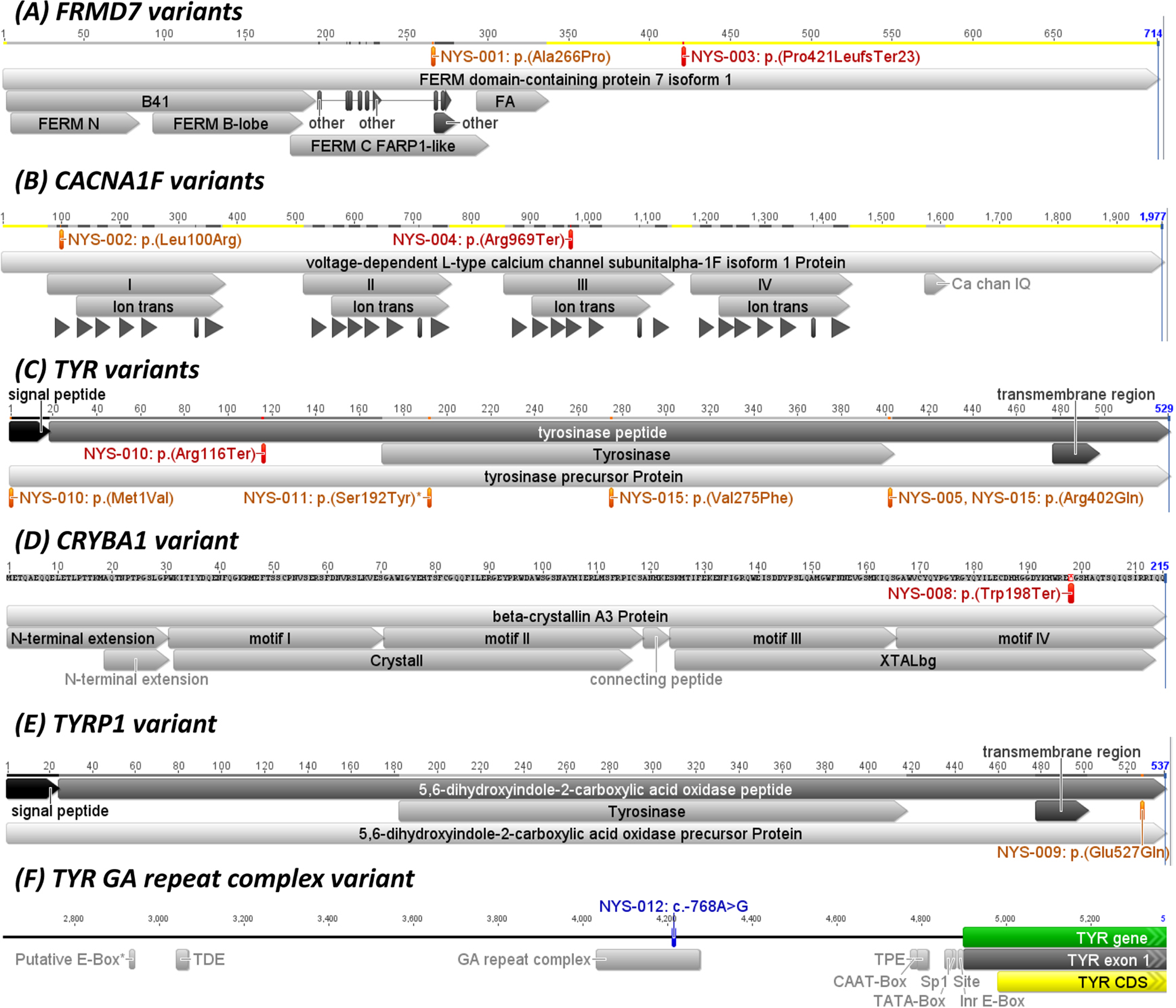

Supplement: Supplementary Figure S3 [file ejhg201744x5.tif]
